# ChatGPT in Medical Education: A Precursor for Automation Bias?

**DOI:** 10.2196/50174

**Published:** 2024-01-17

**Authors:** Tina Nguyen

**Affiliations:** 1 The University of Texas Medical Branch Galveston, TX United States

**Keywords:** ChatGPT, artificial intelligence, AI, medical students, residents, medical school curriculum, medical education, automation bias, large language models, LLMs, bias

## Abstract

Artificial intelligence (AI) in health care has the promise of providing accurate and efficient results. However, AI can also be a black box, where the logic behind its results is nonrational. There are concerns if these questionable results are used in patient care. As physicians have the duty to provide care based on their clinical judgment in addition to their patients’ values and preferences, it is crucial that physicians validate the results from AI. Yet, there are some physicians who exhibit a phenomenon known as automation bias, where there is an assumption from the user that AI is always right. This is a dangerous mindset, as users exhibiting automation bias will not validate the results, given their trust in AI systems. Several factors impact a user’s susceptibility to automation bias, such as inexperience or being born in the digital age. In this editorial, I argue that these factors and a lack of AI education in the medical school curriculum cause automation bias. I also explore the harms of automation bias and why prospective physicians need to be vigilant when using AI. Furthermore, it is important to consider what attitudes are being taught to students when introducing ChatGPT, which could be some students’ first time using AI, prior to their use of AI in the clinical setting. Therefore, in attempts to avoid the problem of automation bias in the long-term, in addition to incorporating AI education into the curriculum, as is necessary, the use of ChatGPT in medical education should be limited to certain tasks. Otherwise, having no constraints on what ChatGPT should be used for could lead to automation bias.

## Introduction

With the introduction of artificial intelligence (AI), automated processes for nearly most tasks have become the norm. In the clinical environment, AI has been used for diagnosis, prognosis, and administrative tasks. Given the popularity of other forms of AI—as seen most recently with ChatGPT, a large language model developed by the company OpenAI—there are suggestions for its potential role in medical education. Users of ChatGPT boast its efficiency and relative accuracy, such as in the generation of a patient’s discharge summary or the conduction of literature reviews [1]. As advancements in medicine continue to arise, medical students are burdened with the impossible task of balancing the need to continuously learn and retain competencies and the need to provide compassionate patient care. As a result, some medical students might feel an incentive to use ChatGPT to save them time in their busy schedules. However, despite the novel acclaim, the technical and ethical issues seen with AI, such as biased results or nonsensical outputs, also plague ChatGPT. These problems become exacerbated when medical students inadvertently develop automation bias, where they overrely on AI, and continue to have this mentality when they become residents, at which point they have the potential to harm patients if the AI provides an erroneous outcome. In this editorial, I argue the justification for AI education in the medical school curriculum and how the lack of it leads to the problem of automation bias, as well as the other harms from automation bias. Subsequently, I connect the implications of students using ChatGPT with automation bias. Finally, I provide recommendations for when ChatGPT use is appropriate.

## The Need for AI Education in the Medical School Curriculum

As the health care landscape has drastically changed through the years, physicians have had to quickly adapt to the digital age. Given the amount of information physicians are required to retain and the new information they must continue to learn, such as information on emerging diseases and the health data of the patients they track, physicians are expected to interact with computer systems in some capacity, whether it is for charting their patients’ information or consulting clinical decision support systems. However, the lack of content on the technological systems in the health care setting inhibits prospective physicians from understanding the benefits of using these technologies, the ethical issues that can arise with their use, and future innovations, along with the wider implications of AI. In Civaner et al’s [2] survey of medical students’ opinions on AI education, they found that 75.6% of students had either limited or no education on the topic of AI. These participants also noted not feeling well equipped to work with AI in the clinical setting. Additionally, in Yun et al’s [3] proposal for future internal medicine physicians, they suggested that these prospective physicians should be able to appreciate the roles of big data and AI in health care. Clearly, there is a desire from students, as well as residency and fellowship programs, to incorporate AI education into the medical school curriculum and training. AI education and training cannot continue to be delayed, as some forms of AI have already been deployed in the clinical setting.

Although several studies have provided proposals for implementing AI education into the medical school curriculum, they have also noted the difficulties of developing AI education, such as schedule constraints and the challenges of deciding the material that should be covered [4,5]. Additionally, this task should not solely be deferred to the attending physicians, as they themselves might not have the adequate training with AI to teach others [5]. Although these challenges serve as barriers to implementing quality AI education into the curriculum, an attempt to include at least some type of education on or educational resources about AI is needed to prepare students and potentially prevent problems in the clinical setting, as further explored in the following section. Therefore, future physicians, medical students, and residents should be trained on the use of AI in health care and other related topics, such as big data or machine learning, to understand the tools they will be working with. Even though medical students should not be expected to be experts in AI and know every technical aspect of these technologies, they should at least feel comfortable with navigating how and when to use AI.

## The Problem of Automation Bias

Although AI is supposed to aid physicians in various processes to decrease their workload and give them more time with their patients, AI can also cause unintended ethical issues. One of the common ethical concerns with AI is that it can essentially be a black box, where the results from the AI are illogical, and the AI developer cannot track how it produced those erroneous results. This problem becomes exacerbated when automation bias arises. Automation bias occurs when a user overrelies on AI systems. Therefore, if a physician exhibits automation bias, then they will not question the results from the AI, potentially leading to bad medical care. In Lyell et al’s [6] study, the error rate associated with a clinical decision support system when it was inaccurate was higher (86.6%) in comparison to the rate it had when it was accurate (58.8%). Although automated processes aid in decision-making and can provide accurate results, there is also the possibility of these systems providing incorrect results and causing irreversible harm on a much larger scale. An example includes the Prescription Drug Monitoring Program (PDMP), a machine learning system that provides risk scores for patients’ likelihood to misuse prescription drugs, which can cause both testimonial injustice and physical harm [7,8]. Testimonial injustice, a form of epistemic injustice, develops when a patient’s account of their health is unfairly dismissed by their physician [8]. Testimonial injustice invalidates the credibility of patients and further implies that their care is dependent on how physicians deem their trustworthiness [8]. A patient’s risk scores can be negatively affected if their chart becomes commingled, which is also known as *overlay*, where a specific person’s electronic health record erroneously pulls in the data of other patients with similar demographic characteristics and compiles these data into 1 chart [7,9]. As such, a patient with chronic pain may not receive the medication they need due to the PDMP providing an incorrect risk score. If a physician uses the risk scores of the PDMP without validating the results or considering their patients’ testimonies, then physical harm, as well as patients’ mistrust toward the physician and the potential deterrence of seeking health care, will ensue. Although AI can aid in the decision-making process, ultimately it is the duty of the physician to ensure that their decisions are based on sound clinical judgment. As such, if a physician with automation bias applies an erroneous outcome to a patient’s care, then the physician becomes accountable for that outcome instead of the AI, as they are the party that used the outcome. To clarify, more sophisticated AI and machine learning systems have been proposed, of which the results would be difficult for users to verify, as these systems use advanced techniques that do not rely on predefined rules. However, the AI systems described in this section are known as *expert systems*, which use a coded set of rules and rely on predefined rules [10]. Even though the verification process might essentially be beyond the scope of some physicians’ expertise regarding future AI and machine learning, physicians should remain attentive to results from AI.

## The Implications for Medical Students and Residents

As seen with the case of the PDMP, automation bias can lead to various harms. Therefore, the systemic issue of automation bias in health care must be addressed. The mentality that AI is always right is often associated with medical students and residents [6,11]. As these groups have grown up in the digital age, they are more comfortable with embracing technology into their practice than older physicians (who either lack digital literacy or are resistant to change). In addition to their openness to using AI, medical students and residents might be prone to automation bias, as they lack experience or are not confident in their skills [11]. Multiple studies have found that algorithmic appreciation—a user’s valuing of an algorithm’s outputs—is lower for users who have more experience in a task than for those who are considered nonexperts in that task [12,13]. A combination of factors, such as newer physicians being digital natives, insufficient expertise, and less overall confidence, highlights how the systemic problem of automation bias came to be. Therefore, the deficiency of AI education in medical school and beyond sets up users to become susceptible to automation bias, as they might be unaware of the technical problems with AI. These users will come into the clinical setting with the assumption that AI systems are always accurate, which will cloud their clinical judgment.

In addition to the broader discussion of AI in health care, which students will inevitably have to interact with at some point in their professional careers, I want to focus on an AI that is accessible to students now—ChatGPT. The fact that ChatGPT has passed the US Medical Licensing Examination could entice students to use ChatGPT [14]. Moreover, Tiwari et al [15], who applied the Technology Acceptance Model to ChatGPT, found that students generally had positive views (in terms of perceived usefulness, credibility, social presence, and hedonic motivation) of ChatGPT based on their previous experiences with using the tool. However, just as AI can be a black-box algorithm, so too can ChatGPT, with respect to its hallucinations. ChatGPT’s hallucinations are results that are seemingly feasible but do not actually exist [1,16]. For example, it is commonly known that ChatGPT can make up citations [16,17]. Additionally, in an editorial, ChatGPT had to be prompted several times by the author to finally respond that it cannot generate visual diagrams [18]. Further, ChatGPT’s data sources only cover data from 2021 and prior years, and as its scope is limited to this context, ChatGPT can provide outdated information [19]. Therefore, despite the acclaim, ChatGPT is not as perfect as some claim it to be. Given the push for ChatGPT use, there is a risk that users might develop an AI solutionism mentality, where users assume that AI has the answer to all problems [10]. AI solutionism is closely related to automation bias, as users with the preconceived notion that AI is always right are more willing to turn to AI. As such, if we train medical students to use ChatGPT, will they be more predisposed to automation bias in the future when they become residents? Although there is no direct answer to this question, given what is known about the medical school curriculum, the context of the student population being composed of digital natives, and the AI solutionism mentality, the possibility of this happening seems likely. Some medical students will take their past, positive interactions with ChatGPT, wherein they received the right response, as confirmation that ChatGPT is reliable. The concern here is that students’ perceptions of the reliability of ChatGPT dictate their views on AI, including AI in the clinical setting, making it easier for them to become susceptible to automation bias. Although some suggest using AI suppression, an approach where an AI’s recommendations are not provided if there is “a higher misleading probability,” to mitigate the risk of automation bias, there appears to be no concrete solutions to solving this problem, especially in the context of the “novice” medical student and resident population [20]. It must also be acknowledged that sometimes, AI use cannot be completely avoided in the health care setting. Thus, in controlling the reoccurrence of automation bias, I believe that students must not only be aware of this potential problem but also build the skills required to prevent this mentality. When addressing the risks of AI in the medical school curriculum, automation bias needs to be a discussion topic. Besides teaching about automation bias, when training medical students, it is important to consider the “hidden curriculum” about using AI, that is, the implied lessons, cultures, and views that students learn in lectures or from observations of faculty [21]. If faculty also fall into the trap of AI solutionism, this will lead to a biased perspective on AI and contribute to the “hidden curriculum.” Faculty should serve as an example for students by ensuring that students have the right critical analysis skills and are comfortable with questioning results instead of accepting what is being given to them. This builds students’ confidence in trusting their instincts, which could deter them from automation bias.

## When Should ChatGPT Be Used in Medical Schools?

Although this editorial takes a more critical stance on AI and ChatGPT, I want to clarify that this does not mean that these tools should never be used or that their functionalities are ineffective. Notably, in the preclinical phase, the medical school curriculum is not catered to students, as the focus is on ensuring that students have expertise on basic medical concepts, the structure and functions of the body, diseases, diagnoses, and treatment concepts [22,23]. This might be a challenge for some students who prefer different learning methods as opposed to the typical didactic method. ChatGPT can be a beneficial tool for students who prefer student-centered or self-directed learning, as it excels in summarizing information and generating practice questions [18,19,24,25]. Students who struggle with a concept in class or want further explanations could also use ChatGPT as an additional resource. Being able to personalize their learning experiences encourages students toward incorporating ChatGPT into their studies. As such, banning the use of ChatGPT could result in students being even more enticed to seek out the “forbidden” chatbot. Therefore, in addition to integrating AI education into the medical school curriculum and avoiding the “hidden curriculum” about AI, students should feel encouraged to use ChatGPT but only to a certain extent.

Despite the advantages of ChatGPT use, students should not be compelled to turn to ChatGPT for every task. For example, assignments that involve students writing about their firsthand experiences would not be appropriate for ChatGPT. With regard to a hypothetical student who delegated such an assignment to ChatGPT, van de Ridder et al [26] stated that “[r]eflections contribute to a learner’s professional development, but this learner robbed themself of an innate self-reflective opportunity.” Students lose a potential outlet for their emotions and the humanistic aspect of care when they delegate ChatGPT to the task of writing a self-reflection piece [27]. Notably, ChatGPT appears to be popular in the context of scientific writing for the following reasons: “efficiency and versatility in writing with text of high quality, improved language, readability, and translation promoting research equity, and accelerated literature review” [1]. However, Blanco-Gonzalez et al [28] argue that “...ChatGPT is not a useful tool for writing reliable scientific texts without strong human intervention. It lacks the knowledge and expertise necessary to accurately and adequately convey complex scientific concepts and information.” There are also concerns about plagiarism with ChatGPT, as it can fabricate citations, fail to disclose all references, and provide inaccurate content (as it only uses information from 2021 and prior years) [1,17]. Therefore, ChatGPT should not be used for writing, as it deprives students of the opportunity to engage in their professional identity and, for those wanting to go into research, the necessary research skills to conduct empirical or conceptual work. Additionally, some web-based educational resources, such as modules or augmented reality, might help supplement students’ experiences during the clinical phase [29]. However, the use of these resources, including ChatGPT, should not be the only learning experience that students have in the clinical phase. In order to build their interpersonal skills and practice humanistic care, students must interact with real patients and other professionals in the clinical setting. Although some students might feel prepared for these interactions (based on their experiences of working through case scenarios that ChatGPT generated for them), they will soon realize that they cannot predict or account for how patients or others (eg, a patient’s family, members of the care team, etc) react in real time. Learning to accommodate patients’ needs and working in a team cannot realistically be achieved with ChatGPT. Instead, these skills are cultivated through students’ experiences in the clinical setting.

The focus should not be on deciding whether to use ChatGPT but on determining the best contexts that ChatGPT can be applied to. As seen in this editorial, ChatGPT excels at particular tasks, such as summarizing information and creating study materials [18,19,24]. Ideally, students should use ChatGPT to supplement their learning experience rather than use it as their sole resource for medical science education. Students should still validate the results (to the extent that they can) from ChatGPT, because it can provide inaccurate results and the problem of hallucinations persists, before they wholeheartedly study or apply the wrong information. When used in this context, ChatGPT plays a lesser role in students’ education, thereby further enhancing their ability to discern results and avoiding AI solutionism.

## Conclusion

To minimize the risk of students developing automation bias, we need to ensure that students receive proper AI education, in which the courses and lessons will teach them about the ethical issues surrounding AI technologies, as well as the problem of automation bias, and encourage the moderate use of AI. ChatGPT should only be used for certain tasks, and it should not be the default resource that students turn to, as this could cause a domino effect, where students develop the automation bias mentality as a result of developing the AI solutionism mentality. Therefore, training medical students to avoid falling into these traps of AI solutionism and automation bias starts in the classroom. Again, the medical school curriculum must reflect the current needs of the students. Furthermore, faculty serve as an example for students; therefore, they should also be proactive in deterring the use of ChatGPT for all tasks and be careful not to contribute to the “hidden curriculum” about AI. Overall, ChatGPT is an assistive tool but only when used in the right context.

## Abbreviations

AI: artificial intelligence
PDMP: Prescription Drug Monitoring Program
